# The Effect of Animate-Inanimate Soundscapes and Framing on Environments’ Evaluation and Predicted Recreation Time

**DOI:** 10.3390/ijerph17239086

**Published:** 2020-12-05

**Authors:** Paulina Krzywicka, Katarzyna Byrka

**Affiliations:** Faculty of Psychology in Wroclaw, SWPS University of Social Sciences and Humanities, Ostrowskiego 30b, 53-238 Wroclaw, Poland; kbyrka@swps.edu.pl

**Keywords:** animate, inanimate, soundscape, frame, label, natural environment, urban environment, recreation

## Abstract

In this research, we investigated whether soundscapes’ animateness and the framing of environments affect participants’ assessment of the surroundings and their predicted recreation time. In an online study, we showed the participants six stimuli, each consisting of an animate or inanimate soundscape recording and of a verbal label of a natural or urban environment. We asked them to (a) imagine visiting the presented locations while mentally fatigued, in company or alone; (b) to visualize spending time there while engaged in recreational activities; and (c) to assess the environment and the predicted recreation time. We found that environments with animate soundscapes were rated as having a higher degree of naturalness and were favored in the urban condition. Environments with inanimate soundscapes, meanwhile, were preferred in the natural condition. Furthermore, natural-framed soundscapes were evaluated as having a higher degree of naturalness and were preferred over urban-framed soundscapes. Social context did not affect the results; however, we discovered the indirect effect of natural labels on the recreation time through the naturalness of the environments, both for the environments with animate and inanimate soundscapes. Overall, our findings demonstrate the influence of soundscapes’ animateness and framing on the settings’ evaluations and on recreation time.

## 1. Introduction

When, in a 2017 documentary entitled “Judi Dench: My Passion for Trees,” the famous British actress uncovered the inner life of trees with the help of a team of experts, she was surprised to hear the sound of water traveling up the trunk and stunned to discover that trees form a community, within which they are able to communicate and cooperate when needed. Astounded by what she had learned, she concluded, “I’ve loved trees all my life, but after this year, I’ll never be able to look at them in the same way again. I shall never be able quite to walk so nonchalantly through a woodland again” (0:56 s.) [1]

The example cited above suggests that animateness—understood as the extent to which an entity is perceived as living or nonliving—can alter one’s impression of the environment, leading to a more favorable evaluation. Earlier research has shown that the human tendency to prefer living organisms is innate [2] and that living things attract more attention [3] or are retained for longer in our memories than nonliving stimuli [4]. However, our understanding of the effect of animateness on the evaluation of our surroundings or our willingness to spend time in such locations remains incomplete: because research hitherto has focused rather on the natural–urban dichotomy, more subtle differentiation between animate and inanimate environments was given much less consideration. Nevertheless, due to the diversity of urban landscapes, with their various levels of animateness, it appears that the presence of living elements has the potential to redefine how urban settings are perceived by boosting their naturalness and thus affecting their evaluation.

In the context of increasing urbanization, which limits people’s direct contact with nature, the demand for effective solutions that will facilitate the creation of more spaces with natural features, particularly within urban areas, is increasing. However, to introduce beneficial changes into these landscapes, more insight is needed into the factors that influence our perceptions of such settings. As such, our study’s objective was to explore the role of animate and inanimate soundscapes in the evaluation of the perceived naturalness of environments and of environmental preferences as well as in the length of recreation time that people are willing to spend in those surroundings. We also aimed to examine the impact of natural and urban frames on these assessments.

## 2. Literature Review

### 2.1. Preferences for Natural Environments and Soundscapes

Human perception of natural and urban landscapes has been studied extensively [5,6,7], demonstrating people’s consistent preference for natural settings. Staats et al. [8], for example, demonstrated that participants who were shown two sets of photographs illustrating either a walking route through a forest or through a city favored the woodland images over the urban scenery and rated the likelihood of restoration and reflection as higher for the natural than for the manmade surroundings. Similarly, Purcell et al. [9] revealed that those who were asked to rate preference and restorative values gave the highest score to natural scenes and the lowest to those representing urban, industrial settings.

Fondness for nature, however, is not limited to natural landscapes. It is also prevalent in the natural sounds and soundscapes that are regarded as auditory equivalents of visual scenery [10]. In previous studies, natural soundscapes have repeatedly been favored over urban ones. Axelsson et al. [11], for example, demonstrated that while road-traffic noise negatively impacted Mariatorget Park’s soundscape quality, natural sounds improved it. Krzywicka and Byrka [12], in turn, found that participants who were asked to take an imagined walk through different locations represented by natural and urban soundscapes favored the former over the latter and reported the walk amid the natural soundscapes to be more restorative.

### 2.2. Evolutionary Perspective and the Biophilia Hypothesis

The explanation for the human tendency to favor natural environments over built-up settings is grounded in the evolutionary perspective. Accordingly, the long history of preference for natural settings is explained by their ability to enhance survival chances associated with resources rich in water and vegetation [13]. Orians [14] argues that the genesis of this preference lies in the similarity of contemporary natural surroundings to the prehistoric savannah—the mosaic-like set of open and wooded habitats of early hominins [15]. Appleton [16], in turn, proposes that humans’ inclination toward natural landscapes is rooted in their ability to provide hideouts and facilitate the detection of potential threats.

The evolutionary explanation evolved into the biophilia hypothesis [2], in which biophilia is defined as the “innately emotional affiliation of human beings to other living organisms” [17] (p. 31). The attraction to nature in this approach is driven not only by people’s biological needs but also by their desire for “aesthetic, intellectual, cognitive, and even spiritual meaning and satisfaction” [18] (p. 20), which can take many forms. It was noticed, for example, that the presence of living beings, such as animals or humans, on landscape paintings receives viewers’ attention [19].

### 2.3. The Significance of the Animate–Inanimate Distinction

The need for interaction with plants and animals reflects humans’ attraction toward “life” [2]. It also indicates the importance of the animate and inanimate distinction, suggesting that living beings may be perceived differently from nonliving forms. Research findings seem to confirm this, demonstrating the presence of the animate–inanimate differentiation from infancy. Molina et al. [20], for instance, demonstrated that infants’ attention was retained for longer when a female research assistant was talking to another person than when she was talking to a ball, whereas Rostad, Yott, and Poulin-Dubois [21] found that 14-month-old children were capable of sorting figurines of humans and animals separately from those of vehicles and furniture.

However, the animate–inanimate distinction extends beyond early development, also affecting cognitive processes such as memory and attention in adult life. Bonin et al. [4] showed, for example, that animate stimuli were remembered better and categorized more quickly than inanimate ones, regardless of whether they were presented in the form of words or pictures. Altman et al. [3], meanwhile, demonstrated that changes in scenes were detected more quickly and more correctly when animals rather than inanimate objects appeared. A similar effect was observed by Giordano et al. [22], who revealed that compared to nonliving environmental sounds (e.g., of a skateboard passing by), living environmental sounds (e.g., of a snorting horse) were identified more quickly and accurately.

### 2.4. The Function of Framing

As noted by Giordano et al. [22], both auditory and verbal stimuli can trigger associations with corresponding environments, providing context for the interpretation of new information. Such effect has been observed in several studies. Haga et al. [23], for example, showed that when the same audio recording—a combination of pink and white noise—was heard by mentally fatigued participants, its reception differed depending on its attributed source. Those who were told that the noise came from a waterfall perceived the sound as more restorative than those who were informed that it emanated from active machinery. Participants from the control condition, meanwhile, who were not notified of the noise’s origin, altered their evaluations according to its ascribed source so that those who believed it had a natural origin assessed the sound as more restorative than those who linked it to an industrial setting.

Descriptions or verbal labels, presented along with the stimulus, can thereby provide frames for the evaluation, influencing a shift of preferences [24]. Such impact of framing on human perception has been repeatedly demonstrated. For instance, Wagner et al. [25] revealed that when photographs with disgusting content (e.g., worms) were framed as “art,” they triggered responses associated with art, leading in consequence, to more positive evaluations of the images. However, when the same photographs were framed as “non-art”, they activated hygiene- and health-related responses instead, contributing to a more negative evaluation.

### 2.5. Goals of the Research

Surprisingly, although the effect of framing was shown to affect people’s judgments, less attention has hitherto been paid to its role in the perception and evaluation of various environments. However, if for some people, urban surroundings can elicit negative associations, such as violence or noise [26], we may assume that the perception of environmental stimuli can be adversely affected by such impressions of built-up areas. By contrast, if natural environments activate positive associations, such as recreation and leisure, the stimulus perceived as natural will be evaluated favorably.

Further consideration should also be given to the role of the animate–inanimate distinction in the perception of environments, as its impact on people’s behaviors and evaluation of natural and urban surroundings has not been sufficiently examined, particularly with respect to people’s desire for affiliation with animate environments.

This study’s main aim was to explore the impact of soundscapes’ animateness and of natural and urban frames on the evaluation of perceived naturalness and environmental preferences as well as on the assessment of predicted recreation time spent in such surroundings. As we wished to gain greater insight into factors with the potential to influence the participants’ assessment of the environments, we also decided to investigate the role of social context. This was for two reasons: first, because it had previously been analyzed alongside the type of environment and was shown to affect environmental preferences [12,27], and second, to test whether the presence of others would disturb the participants’ perception of the environments’ animateness.

We expected that soundscape recordings framed as natural or urban environments would lead to differences in their interpretation and evaluation. In line with existing research, we hypothesized that environments represented by soundscapes with natural labels would be assessed as having a higher degree of naturalness than those presented with urban labels (Hypothesis 1). Moreover, because of the tendency observed among children to define nature as “living things” [28], we predicted that the perceived naturalness of the environments would decrease with the decline in the soundscapes’ animateness, both with natural (Hypothesis 2a) and urban frames (Hypothesis 2b).

As humans’ preference for nature has been well-documented [5,6,29], we expected participants to prefer environments labeled as natural to those labeled as urban (Hypothesis 3). Based on the findings that people who are in a state of mental fatigue prefer solitude over being in company [30], we also hypothesized that imagined mental fatigue would cause participants to prefer environments that they visited alone to those visited in the company of others (Hypothesis 4). Furthermore, due to the seemingly inborn inclination toward living beings [2], we predicted that environmental preferences would increase with the elevation of the soundscapes’ animateness in both natural (Hypothesis 5a) and urban conditions (Hypothesis 5b).

Considering that hinting at the purpose of visiting a given location has been shown to affect people’s mental representation of the behavior or actual behavior expected in such a setting [31], we also assumed that participants, asked to imagine spending time on recreation, would dedicate various lengths of time depending on the level of naturalness depending on the attributed natural or urban frames. Thus, we hypothesized that we would find the indirect effects of natural environments on predicted recreation time through the perceived naturalness of environments for environments presented with both animate (Hypothesis 6a) and inanimate (Hypothesis 6b) soundscapes.

## 3. Materials and Methods

### 3.1. Design

The study was conducted according to a 2 × 2 × 2 design with the environment (natural vs. urban labels) and social context (being alone vs. in company) as between-subject factors and the soundscapes’ animateness (animate vs. inanimate) as within-subject factors.

### 3.2. Participants

Two hundred and fifty-eight Amazon MTurk employees of high reputation (above 95% approval ratings, 1000 or more completed Human Intelligence Tasks—HITs; [32]) visited the study website. Forty-two of these were excluded from further analyses, either due to multiple and/or overlapping log-in attempts (*n* = 38) or because they did not pass the sound-check test (*n* = 4). Of the remaining 216 participants, 49% were men and 51% were women, aged between 21 and 72 years (*M* = 40.20, *SD* = 11.26). The majority were White (78%) and came from North America (98%). Most participants (99%) identified English as their first language.

### 3.3. Procedure

The research was conducted online. Participants read a short description of the study on the Amazon MTurk platform and clicked on the provided link, which redirected them to the website, where the experiment was administered using Inquisit 5 Web [33].

After signing an informed consent form, they were asked to imagine themselves in a state of mental fatigue and to complete a short scale evaluating the impact of the scenario. Next, they were requested to turn on their computing devices’ speakers. To verify whether they complied with the requirement, a number (“number three”) was read aloud in a man’s voice (twice) and the participants were asked to type it in the text box provided. Six different stimuli were then displayed in a random order, each consisting of a 30 s long soundscape recording (e.g., of the sound of swimming) and of the matching verbal label framing, depending on the randomly assigned condition—a natural (e.g., a lake) or an urban environment (e.g., a swimming pool). Each presentation was preceded by a short instruction, in which participants were asked to imagine (a) visiting given environment while in a state of mental fatigue and (b) spending some time there engaged in recreational activities that they would enjoy. Half of the participants were randomly assigned the scenario of being in the location alone, while the other half were assigned the scenario of being there in the company of friends, loved ones, or family members. Following each display, the participants assessed the naturalness of the environment, their environmental preferences, and the predicted length of recreation time that they would spend in the given settings. When all presentations and evaluations had been completed, the participants answered additional questions (e.g., about the equipment used, demographics) and were informed of the experiment’s aim. They were also provided with the researcher’s e-mail address in case any questions concerning the study should arise.

The entire procedure, approved by the Faculty Ethics Review Board (06/P/04/2020), lasted on average 54 min. Those who finished the research and fulfilled its obligatory condition (had the speakers turned on) were paid $3 for their participation.

### 3.4. Materials and Measures

All materials and measures were programmed using Inquisit 5 Web [33]. The same software was used for data collection.

#### 3.4.1. Mental Fatigue Scenario

Owing to the ubiquity of fatigue in the population [34], we aimed to control participants’ state of mental fatigue. However, because the procedure of our study required longer time than an average MTurk task [35], we decided to rely on participants’ imagination, instead of creating an objective mental fatigue manipulation. Our decision was encouraged by previous research, showing that mental fatigue scenarios were found to be effective [8,12,27]. Thus, we asked the participants to imagine themselves feeling stressed, negative, and mentally exhausted, such as after an intense period of work or study, when problems with concentration and irritation arise, thereby replicating the vignette described by Ratcliffe and Korpela [36]. Although we requested that the participants keep this scenario in mind throughout the entire study, they were reminded of it prior to each display of the environmental stimulus.

#### 3.4.2. Social Context Scenario

Depending on the random assignment, half of the participants were instructed to visualize themselves spending time engaged in recreation in the imagined settings in the company of someone with whom they are well acquainted—for example, friends, loved ones, or family members—while the other half were asked to imagine being in those places alone, without anyone they know. The scenario preceded each presentation of the environmental stimulus.

#### 3.4.3. The Environment

We prepared twelve different verbal labels (six per condition) to frame the soundscapes as “natural” or “urban” environments. Each label introduced a general concept associated with a given location and was displayed on a screen with a matching soundscape that was played through headphones or speakers (see Table 1). The order in which the labels were presented was random.

#### 3.4.4. Animateness of the Soundscapes

To represent the soundscapes of the environments for which the verbal labels were shown to the participants, six WAV audio files (see Table S1 in Supplementary Materials) of the opposite animateness level were selected from the BBC Sound Effects and Field Recordings Library [37]. Half of these included sounds of animals’ vocalizations and movements (animate soundscapes), while the other half did not include vocal sounds of any living organisms, but as in the study of Giordano et al. [22] consisted of sounds generated by environments and/or by living agent’s activities (inanimate soundscapes).

Additional selection criteria included the absence of mechanical noise or human voice and openness to interpretation, as depending on the assigned label the recordings were supposed to represent locations in either the natural or urban environments (see Table 1). Based on the findings by Gygi et al. [38], chosen stimuli should have been familiar to participants.

Each audio file was shortened to 30 s, converted into an MP3 file, and paired with a natural or urban label.

#### 3.4.5. Effect of the Mental Fatigue Scenario

After reading the mental fatigue scenario, participants were asked how tired, stressed, negative, and mentally worn out they would have felt in the described situation. They provided their responses on a 7-point Likert-type scale ranging from 0 (Not at all) to 6 (Completely), and the total score was obtained by averaging all the items. The scale (α = 0.93), with a mean of 3.89 (*SD* = 1.61), was inspired by manipulation checks used by Staats et al. [8] and by the mental fatigue scenario, based on the vignette described by Ratcliffe and Korpela [36].

#### 3.4.6. Perceived Naturalness of the Environments

The perceived naturalness of the environments was assessed using visual analog scales (six per condition), inspired by the one-item scale employed by Berman et al. [39]. Following each presentation of the stimulus, we requested participants to describe the environment by placing a mark on a 10-cm-long line, with anchors ranging from Urban (0 points) to Natural (100 points). The total scores were achieved by averaging the answers separately for environments presented with animate (α = 0.74) and inanimate (α = 0.78) soundscapes.

#### 3.4.7. Environmental Preferences

The measure of environmental preferences was constructed based on the items described in the study by Staats et al. [8]. Participants were asked to indicate on a scale from 1 (Not at all) to 7 (Completely) the extent to which they considered each of the presented environments to be beautiful, nice, and pleasant. The answers were then averaged separately for the environments with animate (α = 0.61) and inanimate soundscapes (α = 0.63).

#### 3.4.8. Recreation Time

Visual analog scales were again used to rate how much time participants would spend engaged in recreation in each of the given environments. The predicted amount of time was indicated by selecting a point on a 10 cm line with the left anchor described as 0 min and the right anchor marked as 100 min or more. To ensure that the participants clearly understood the concept of recreation, a short definition of the term (“recreation is defined as a form of activity, done for pleasure or amusement, when one is not working”), based on the Longman Dictionary of Contemporary English Online [40] and on the Cambridge Dictionary [41], was displayed prior to the visual analog scale instruction. The total recreation time for all environments was calculated by averaging the provided responses (α = 0.67). Cronbach’s alpha for the environments with animate soundscapes equaled 0.59, while for those presented with inanimate soundscapes, it was 0.54.

#### 3.4.9. Questions about the Equipment and Demographics

In addition, we collected information about the type of equipment (a smartphone, a tablet/iPhone, a PC, a laptop/notebook, or other) and the kind of speakers (earphones, headphones, built-in speakers, or other) used by participants. However, since they are not the direct subject of our research and, additionally, they had no influence on our findings, they were not analyzed in this paper. We also asked demographic questions (e.g., age, gender).

## 4. Results

### 4.1. Changes in the Perceived Naturalness of the Environments

Despite the violation of the assumption of homogeneity of variance for inanimate soundscapes (*p* = 0.001), we continued the analysis based on the presumption that when sample sizes are close to equal, ANOVA is quite robust to heterogeneity of variance [42]. A 2 × 2 × 2 mixed ANOVA was thus performed to analyze whether changes in the framing of the environments, social context, or soundscapes’ animateness influenced the perceived naturalness of the presented environments. The environment (natural or urban labels) and social context (being alone or in company) were entered as between-subject variables, while animateness of the soundscapes (animate or inanimate) served as a within-subject variables, with perceived naturalness of the environments as dependent variables.

The analyses yielded a significant strong main effect of the environment, *F*(1, 168) = 158.93, *p* < 0.001, partial η² = 0.49. As predicted (Hypothesis 1), participants in the natural condition assessed the environments as having a higher degree of naturalness (*M* = 77.50, *SD* = 14.96) than those in the urban condition (*M* = 49.18, *SD* = 19.28). Nevertheless, although they preferred being alone (*M* = 64.97, *SD* = 17.53) to being with someone (*M* = 61.71, *SD* = 16.71), the main effect of social context was non-significant, *F*(1, 168) = 2.10, *p* = 0.149, partial η² = 0.01. There was, however, a significant main effect of animateness of the soundscapes, *F*(1, 168) = 258.10, *p* < 0.001, partial η² = 0.61, as environments presented with animate soundscapes were perceived as having a higher degree of naturalness (*M* = 74.77, *SD* = 16.44) than those shown with inanimate soundscapes (*M* = 51.91, *SD* = 17.80).

The two-way interaction of the environment and social context was non-significant, *F*(1, 168) = 0.06, *p* = 0.813, partial η² < 0.001. Nonetheless, we found a significant two-way interaction between the soundscapes’ animateness and the environment, *F*(1, 168) = 68.46, *p* < 0.001, partial η² = 0.29, confirming Hypotheses 2a,b. Although in both the natural and urban conditions the perceived naturalness of the environments declined as the soundscapes’ animateness decreased (from *M* = 83.04, *SD* = 15.98 to *M* = 71.96, *SD* = 13.94 in the natural condition and from *M* = 66.50, *SD* = 16.90 to *M* = 31.87, *SD* = 21.66 in the urban condition), the decline was more pronounced for those to whom the urban labels were shown, suggesting that the interaction was driven by low evaluations of naturalness in the urban and inanimate conditions.

**Figure 1:**
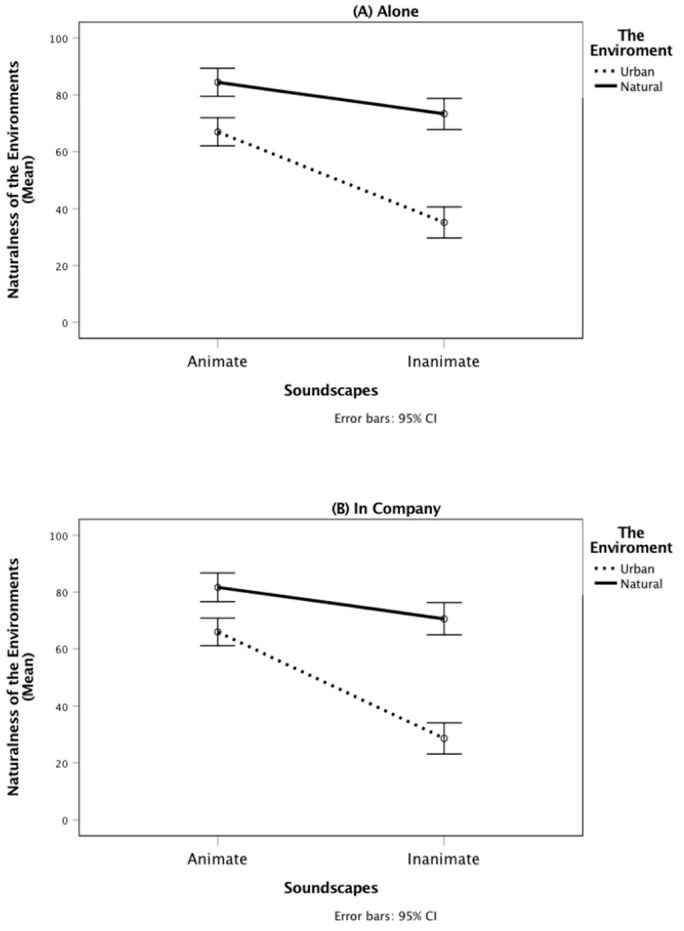
The interaction between animateness of the soundscapes and the environment on naturalness of the environments for the “Alone” (**A**) and “In Company” (**B**) conditions.

The two-way interaction between animateness of the soundscapes and social context, *F*(1, 168) = 0.92, *p* = 0.338, partial η² = 0.01, was non-significant, so also was the three-way interaction between animateness of the soundscapes, the environment, and social context, *F*(1, 168) = 0.97, *p* = 0.327, partial η² = 0.01 (see Figure 1).

### 4.2. Changes in Environmental Preferences

A 2 × 2 × 2 mixed ANOVA was conducted once more to investigate whether changes in the framing, social context, or animateness of the presented soundscapes had an effect on participants’ environmental preferences. As previously mentioned, the environment (natural or urban labels) and social context (being alone or in company) were entered as between-subject variables, and animateness of the soundscapes (animate or inanimate) was included as a within-subject variable. Environmental preferences served as dependent variables.

We found a significant main effect of the environment, *F*(1, 168) = 8.37, *p* = 0.004, partial η² = 0.05, which showed that, as hypothesized (Hypothesis 3), participants had stronger preferences for environments with natural (*M* = 5.39, *SD* = 0.98) rather than urban (*M* = 5.00, *SD* = 0.97) labels. Contrary to our predictions (Hypothesis 4), the main effect of social context was non-significant, *F*(1, 168) = 1.16, *p* = 0.282, partial η² = 0.01. The main effect of animateness of the soundscapes, *F*(1, 168) = 0.05, *p* = 0.833, partial η² < 0.001, and the two-way interaction between the environment and social context, *F*(1, 168) = 0.10, *p* = 0.758, partial η² = 0.001, were also non-significant.

There was, however, a significant two-way interaction between the animateness of the soundscapes and the environment, *F*(1, 168) = 22.53, *p* < 0.001, partial η² = 0.12. While the participants from the natural condition showed increased environmental preferences as the animateness of the soundscapes decreased (from *M* = 5.55, *SD* = 0.89 to *M* = 5.22, *SD* = 1.06), the opposite pattern was found for participants from the urban condition, for whom environmental preferences decreased as the animateness of the soundscapes decreased (from *M* = 5.15, *SD* = 1.02 to *M* = 4.85, *SD* = 0.91). Thus, although Hypothesis 5a was not supported, Hypothesis 5b was confirmed.

**Figure 2:**
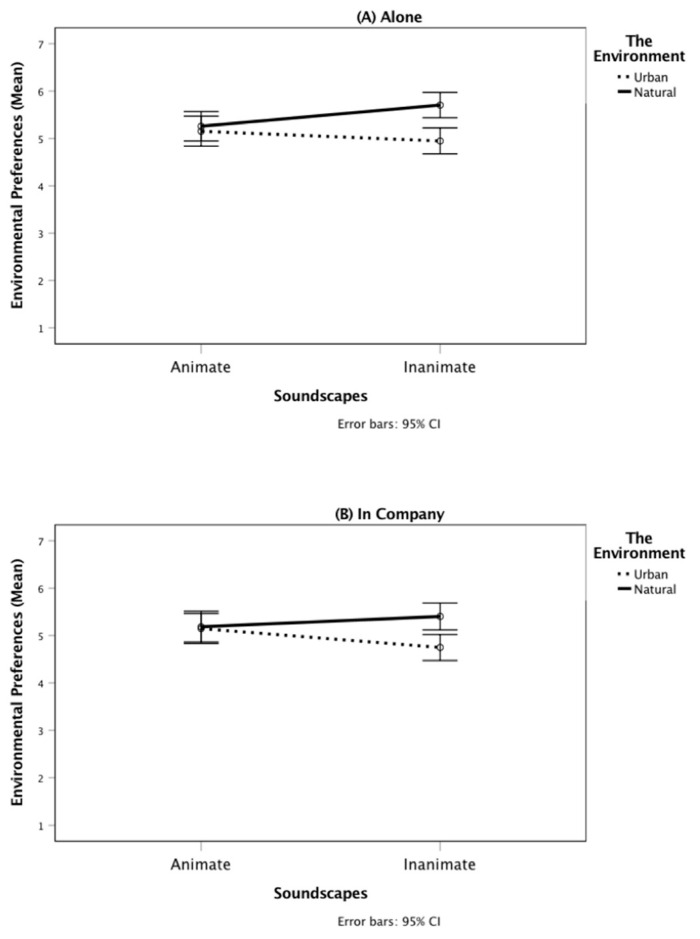
The interaction between animateness of the soundscapes and the environment on environmental preferences for the “Alone” (**A**) and “In Company” (**B**) conditions.

A similar trend was found in the interaction between animateness of the soundscapes and social context: for those from the “alone” condition, environmental preferences increased as the soundscapes’ animateness decreased, while for those who imagined being in company, environmental preferences decreased as the soundscapes’ animateness decreased; however, the interaction was non-significant, *F*(1, 168) = 2.59, *p* = 0.109, partial η² = 0.02. As was the interaction between animateness of the soundscapes, the environment, and social context, *F*(1, 168) = 0.02, *p* = 0.891, partial η² < 0.00 (see Figure 2).

### 4.3. Indirect Effect of the Environment on Recreation Time via Naturalness of the Environments for Environments with Animate Soundscapes

**Figure 3:**
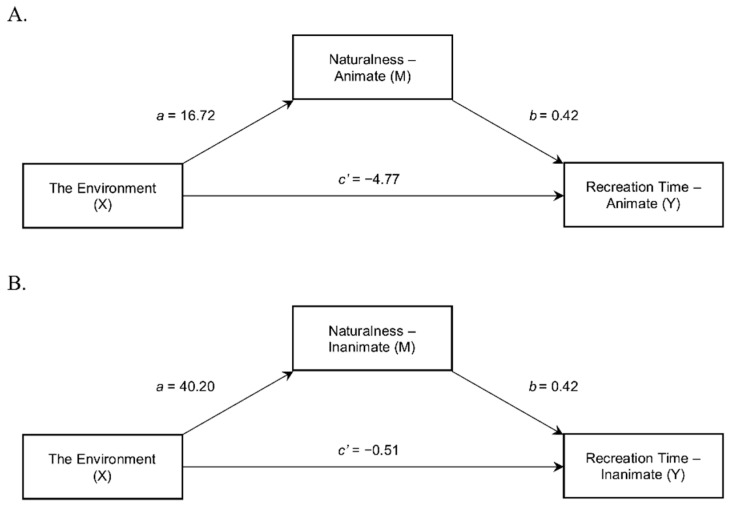
Indirect effect of the environment on recreation time via the naturalness of the environments for environments with animate (**A**) and inanimate soundscapes (**B**)—statistical diagrams containing regression coefficients.

Hayes’ [43] PROCESS Macro (Model 4) with 5000 bootstrap samples and 95% confidence intervals was used to examine the indirect effects of the environment (X) on the predicted recreation time (Y) through the perceived naturalness of the environments (M) for the environments presented with animate soundscapes (see Figure 3A). A heteroscedasticity-consistent standard error approach (HC3) was also applied, as recommended by Hayes and Cai [44] and Hayes [43].

As expected (Hypothesis 6a), we found a significant indirect effect of the environment on recreation time through the perceived naturalness of the environments (*ab* = 6.96, 95% CI: [3.73, 10.89]) for environments presented with animate soundscapes. When the labels of natural rather than urban environments were shown to the participants, the perceived naturalness of the environments with animate soundscapes was boosted (*a* = 16.72), and the increased perceived naturalness of environments with animate soundscapes translated into prolonged recreation time in those environments (*b* = 0.42). No evidence was found to indicate that the environment influenced predicted recreation time independent of its effect on the perceived naturalness of the environments (*c’* = −4.77, *p* = 0.197). Model coefficients for the analysis can be found in Table 2.

### 4.4. Indirect Effect of the Environment on Recreation Time via Naturalness of the Environments for Environments with Inanimate Soundscapes

PROCESS Macro (Model 4) [43] with 5000 bootstrap samples and 95% confidence intervals was performed again to test the indirect effects of the environment (X) on the predicted recreation time (Y) via the perceived naturalness of the environments (M) for environments presented with inanimate soundscapes (see Figure 3B). As previously, the heteroscedasticity-consistent standard error approach (HC3) was applied.

A significant indirect effect of the environment on the recreation time via the perceived naturalness for environments with inanimate soundscapes was observed (*ab* = 16.87, 95% CI: [11.26, 23.04]), which confirmed Hypothesis 6b. Participants who saw the natural labels assessed the environments with inanimate soundscapes as having a higher degree of perceived naturalness than those to whom the urban labels were displayed (*a* = 40.20), and the increase in the perceived naturalness of those environments led to the extension of the predicted recreation time in environments with inanimate soundscapes (*b* = 0.42). No evidence was found to suggest that for the environments with inanimate soundscapes, the environmental labels influenced recreation time independent of their effect on the perceived naturalness of the environments (*c’* = −0.51, *p* = 0.898). For model coefficients, see Table 3.

## 5. Discussion

In this study, we examined factors contributing to the evaluation of the perceived naturalness of environments, environmental preferences, and predicted recreation time. Our focus was on the significance of the soundscapes’ animateness and on the role of natural and urban frames. However, we also analyzed the influence of social context.

We found that natural and urban labels had an impact on the perception of the environments’ naturalness, so that the natural-framed soundscapes were evaluated as having a higher degree of naturalness than those framed as urban. Because the audio recordings were identical in both conditions, it appears that the difference in the assessment can be attributed to the contrasting labels of the presented settings. Since it was shown that the perceived naturalness of the environment depends on its attributed level of human modification, such that the image of a non-modified setting was viewed as more natural than that which was believed to have been altered through anthropogenic activity [45], it may be assumed that urban localizations were more associated with landscapes’ transformations than with natural features. Natural spots, meanwhile, could have been linked with more natural elements and, perhaps, fewer settings’ alterations. Consequently, the perceived naturalness of the environments was higher in the natural and lower in the urban conditions.

The assessment of the perceived naturalness was also found to be affected by the soundscapes’ animateness, because the environments with animate soundscapes were rated as having a higher degree of naturalness than those with inanimate soundscapes. Furthermore, although environments with inanimate soundscapes were perceived as having a lower degree of naturalness, both in the natural and urban conditions, the decline in naturalness was particularly apparent with the display of the urban labels. These findings, in line with the biophilia hypothesis [2,17], confirmed the importance of animateness in the evaluation of the surroundings, as animal sounds were shown to increase the perceived naturalness of the settings in both natural and urban conditions. Interestingly, the influence of the animate soundscapes was significantly more profound in the urban than in the natural condition, which suggests that the presence of living things is more appreciated in built-up areas. While nature itself can be seen as full of life, human-altered settings may often lack the sense of “aliveness.” It appears, however, that animate soundscapes can improve the quality of urban environments by increasing their perceived naturalness. The social context of being in company or alone did not influence the evaluation, revealing that the perceived naturalness of environments was not affected by the presence of others.

We also examined the potential determinants of environmental preferences. Again, we discovered the impact of framing on the settings’ evaluations, as natural-labeled environments were found to be preferred over urban-labeled ones. Thus, the well-established preference for natural settings [5,6] was demonstrated once more. Because participants were asked to imagine themselves to be in a state of mental fatigue, these results can be interpreted in terms of their need for psychological restoration, in line with the Attention Restoration Theory [6] and the Stress Recovery Theory [5], both of which emphasize the restorative potential of nature. These findings, however, can also be attributed to participants’ expectations of presented environments. In such circumstances, rather than being based on audio stimuli, their evaluations may have been based on the history of their previous encounters with natural and urban surroundings, in favor of the former. Thus, the obtained results highlight the capacity of labels to impact the assessment of environments, indicating their potential to a priori bias the settings’ perception and, perhaps, to indirectly influence people’s environmental attitudes and behaviors. Nonetheless, the evolutionary explanation, favoring natural habitats, can also be considered, as the soundscapes selected for the study provided non-threatening environmental cues [29].

People in a state of mental fatigue prefer to be alone [30], but although the mental fatigue scenario was presented prior to each stimulus assessment, contrary to our predictions, environments visited alone were not preferred to those visited in the company of others. One probable explanation of that outcome can be attributed to the labels of the presented environments. It can be assumed that when the soundscapes were associated with concrete activities, such as swimming or horse riding, which participants did not recognize as requiring social contact to be enjoyable, the presence of others did not alter their environmental preferences. If, however, some other spots had been presented, such as a café or a concert hall, the social context may have affected the results, because such places demand more social interaction and are usually visited in company. The other possibility, as indicated by the findings of the study by Krzywicka and Byrka [12], is that the presence of others would have been more significant, if we had asked participants to imagine themselves in a state of relaxation. The results obtained revealed that the imagined social context did not interact with the animateness of the soundscapes. It can be assumed, therefore, that the imagined company may have been perceived independently of the setting and, consequently, did not affect the evaluation.

We also found that the animateness of the soundscapes had no impact on preferences unless in interaction with the environment. Surprisingly, in the natural condition, participants tended to prefer environments with inanimate soundscapes, while in the urban condition, they favored environments with animate soundscapes. It appears that in the natural settings, the presence of animate things was not required to boost environmental preferences. Inanimate soundscapes of natural environments (e.g., of a lake or of a waterfall), meanwhile, may have been associated with corresponding landscapes. If so, they may have created in the participants the sense of being away [6], leading to their more favorable evaluation as they represented locations less commonly encountered in everyday life. In the case of the built-up settings, the opposite pattern was revealed and, in line with the biophilia hypothesis [2,17], animal sounds enhanced environmental preferences, highlighting the significance of animateness with respect to the perception of urban spaces.

Apart from exploring the roles of different factors in the perception and evaluation of the environments, we also examined whether and how these assessments would translate into participants’ behaviors. We observed an indirect effect of the labels of natural environments on the predicted recreation time through the perceived naturalness of the environments, for both the environments with animate and inanimate soundscapes. The increase in the evaluation of the perceived naturalness of environments led to the declaration of longer recreation time; interestingly, however, the mediation effect was stronger for environments with inanimate soundscapes. These findings again highlight the importance of the framing of the environments as well as of soundscapes’ animateness, demonstrating their impact not only on the perception of the settings but also on the possibility of future behaviors in those surroundings. Due to the well-established preference for natural environments [5,6,7], particularly those that have not been anthropogenically altered [45], it may be assumed that when the presented stimuli along with the displayed scenario evoked memories of recreational activities, participants were more likely to imagine themselves spending more time on recreation in the settings, which they perceived as having a higher degree of naturalness, than in those viewed as more influenced by humans. The fact that for environments with animate soundscapes the predicted recreation time appeared to be less dependent on the perceived naturalness can probably be attributed to the sonic cues provided. Since humans feel an innate connection with living creatures [17], it is possible that as a result of hearing the animal sounds, their estimation was less dependent on the imagined naturalness of those environments and thus more influenced by the audio stimuli. For the environments with the inanimate soundscapes, meanwhile, no acoustic evidence of living beings was presented, and the predicted recreation time seemed to be based on the perceived naturalness of similar surroundings, rooted more in the label-based expectations of those settings than in the presented soundscapes.

This study had several limitations that should be mentioned. Because the research was conducted online, we could not control the equipment used by the participants or the settings in which the experiment was performed. Thus, although the choice of Inquisit [33] assured us that participants would not be able to browse the Web while taking part in the study, other circumstances, which may have impacted the results, should also be considered—for example, if the participant was sharing a room with someone despite being in the “alone” condition.

Another issue that must be mentioned is the moderate reliability of some scales (e.g., of environmental preferences or recreation time). Because we designed an online experiment, our intention was to simplify the procedure; yet, by introducing different separation criteria (soundscapes’ animateness instead of the type of environment in the measure of environmental preferences) and by reducing the number of items in the measures, we contributed to a decrease in alpha coefficients [46]. Finally, the fact that we asked participants to imagine the state of mental fatigue, company, or the environments can also be viewed as a limitation. However, to examine the influence of natural and urban labels, rather than using suggestive visual images, we decided to use verbal frames and soundscapes, which seemed more open to interpretation and were shown to be effective [23].

It would be interesting, however, in future research to adopt a more multimodal approach and to study the impact of other senses on the evaluation of environments, using, for example, olfactory stimuli. The elimination of the potential drawbacks of this online experiment, by replicating it in a laboratory setting, among a more diverse sample, and with the use of more complex measures, would also be beneficial. Similarly, further examination of the function of animateness and the framing of the environments in the perception of surroundings but with a greater attention shift toward cultural differences in addition to the landscapes’ and soundscapes’ aesthetics is recommended.

It seems that several practical implications can be drawn from this research. To build a more sustainable world, we must continuously seek methods of implementing natural elements into urban environments to increase the perceived naturalness of the built-up areas, not only with the use of greenery but also by exploiting the potential of animals and their vocalizations. Thus, while designing attractive and citizen-friendly spaces within the urban agglomerations, architects and urban planners should consider introducing more diverse forms of flora (e.g., sensory gardens) and various fauna species into the project. Moreover, whenever space or funds are lacking—for example, for creating a pocket park—even the smallest enhancement, such as erecting bird feeders or bird houses to attract birds, could make a difference in how the setting is perceived. Animate sounds, such as birdsongs, regardless of whether natural or prerecorded, could also be used in frequently visited places to mask unwanted noise (e.g., traffic sounds) or to mark recreational areas. Additionally, more attention should be paid to building animal-friendly municipal infrastructure, allowing humans to enjoy the benefits of contact with living things.

Moreover, to promote change in people’s lifestyles and to boost their subjective well-being, greater effort should be invested in the development of programs aimed at improving the physical appearance as well as the mental image of urban environments. In particular, among children and teenagers, as the research shows that early experiences with environments have an impact on adults’ preferences and outdoor recreational activities [47]. Furthermore, since labels suggesting natural or urban environments can affect the setting’s evaluation, perhaps the naming of places should deserve more consideration as well.

## 6. Conclusions

As stated by Seto et al. ([48], p. 16,083), “if current trends in population density continue and all areas with high probabilities of urban expansion undergo change, then by 2030, urban land cover will increase by 1.2 million km^2^, nearly tripling the global urban land area circa 2000.” In such circumstances, when the environments’ transformation from nonurban to urban seems inevitable, the search for applicable solutions to improve the quality of urban citizens’ life and their surroundings is constantly required.

In this study, we showed that animate and inanimate soundscapes as well as the framing of the environments can have an impact both on the surroundings’ assessment and on the predicted recreation time. There remains, however, much to discover.

## Figures and Tables

**Table 1:** Recordings and verbal labels used in the study.

Soundscapes	The Environment	
Animate	Natural Labels	Urban Labels
A trotting and snorting horse	The American Discovery Trail–a bridle path (horses)	An indoor riding arena (horses)
Singing birds	The Mudumalai National Park and Wildlife Sanctuary in India (birds: mynahs, barbets, parakeets, bulbuls)	An exotic bird exhibition at Bhavan’s College in India (birds: mynahs, barbets, parakeets, bulbuls)
Running and bleating goats	A meadow (goats)	A zoo (goats)
Inanimate		
Falling water	Mountains–a waterfall	An old town square–a fountain
Ice skating	An outdoor ice rink in a park	An indoor ice rink in a mall
Swimming	A lake	A swimming pool

**Table 2:** Model coefficients for the simple mediation for environments with animate soundscapes.

Antecedent	Consequent							
	Naturalness (*M*)				Recreation Time (*Y*)			
		Coeff.	*SE* ^a^	*p*		Coeff.	*SE* ^a^	*p*
The Environment (*X*) ^b^	*a*	16.72	2.48	<0.001	*c’*	−4.77	3.68	0.197
Naturalness of Environments (*M*)					*b*	0.42	0.10	<0.001
Constant	*i_M_*	66.32	1.78	<0.001	*i_Y_*	25.43	7.17	0.001
		*R*² = 0.21				*R*² = 0.09		
		*F*(1, 173) = 45.52 ^a^, *p* < 0.001				*F*(2, 172) = 8.48 ^a^, *p* < 0.001		

Note. ^a^ HC3 corrected. ^b^ 0 = urban environment; 1 = natural environment.

**Table 3:** Model coefficients for the simple mediation for environments with inanimate soundscapes.

Antecedent	Consequent							
	Naturalness (*M*)				Recreation Time (*Y*)			
		Coeff.	*SE* ^a^	*p*		Coeff.	*SE* ^a^	*p*
The Environment (*X*) ^b^	*a*	40.20	2.79	<0.001	*c’*	−0.51	4.01	0.898
Naturalness of Environments (*M*)					*b*	0.42	0.07	<0.001
Constant	*i_M_*	32.03	2.33	<0.001	*i_Y_*	31.79	3.09	<0.001
		*R*² = 0.55				*R*² = 0.30		
		*F*(1, 172) = 207.50 ^a^, *p* < 0.001				*F*(2, 171) = 35.68 ^a^, *p* < 0.001		

Note. ^a^ HC3 corrected. ^b^ 0 = urban environment; 1 = natural environment.

## Supplementary Materials

The following are available online at https://www.mdpi.com/1660-4601/17/23/9086/s1, Table S1: The Links to the Audio Files from the BBC Sound Effects and Field Recordings Library.
