# Supplementary material for: The Effect of Animate-Inanimate Soundscapes and Framing on Environments’ Evaluation and Predicted Recreation Time
Source: Int J Environ Res Public Health. 2020 Dec 5;17(23):9086. doi: 10.3390/ijerph17239086 (PMC7730829; doi:10.3390/ijerph17239086)
Supplement: Supplementary file 1 [file ijerph-17-09086-s001.pdf]

Table S1. The Links to the Audio Files from the BBC Sound Effects and Field Recordings Library.

Soundscapes	Description	Link
Falling water	"Cascade of numerous small waterfalls."	http://bbcsfx.acropolis.org.uk/?q=waterfall
Swimming	"Outdoor swimming pool, swimmer enters water, breast stroke…"	http://bbcsfx.acropolis.org.uk/?q=outdoor+swimming
Ice skating	"Ice Skating, one person circling close…"	http://bbcsfx.acropolis.org.uk/?q=ice+skating
A trotting and snorting horse	"One horse trots past through grass, right to left."	http://bbcsfx.acropolis.org.uk/?cat=horses&q=grass
Singing birds	"Birds at watering hole, Madumbalai National Park…"	http://bbcsfx.acropolis.org.uk/?q=madumbalai+national+park
Running and bleating goats	"Goats mating, with other goats bleating in background…"	http://bbcsfx.acropolis.org.uk/?q=goat
